# Dementia Deaths Most Commonly Result from Heart and Lung Disease: Evidence from the South Carolina Alzheimer’s Disease Registry

**DOI:** 10.3390/biomedicines13061321

**Published:** 2025-05-28

**Authors:** Daniel A. Amoatika, John R. Absher, Md Tareq Ferdous Khan, Maggi C. Miller

**Affiliations:** 1 Department of Epidemiology and Biostatistics, University of South Carolina, Columbia, SC 29208, USA; chandlmj@email.sc.edu; 2 Office for the Study of Aging, University of South Carolina, Columbia, SC 29208, USA; 3 Brain Health Network, University of South Carolina, Columbia, SC 29208, USA; absher@mailbox.sc.edu; 4 Division of Neurology, Department of Medicine, School of Medicine Greenville, University of South Carolina, Greenville, SC 29605, USA; 5 School of Health Research, Clemson University, Clemson, SC 29634, USA; mtkhan@clemson.edu; 6 Department of Public Health Sciences, College of Behavioral, Social and Health Sciences, Clemson University, Clemson, SC 29634, USA

**Keywords:** neurodegeneration, cardiovascular disease mortality, Alzheimer’s Disease and related dementias, mortality risk, underlying causes of death, South Carolina Alzheimer’s Disease Registry, chronic obstructive pulmonary disease

## Abstract

**Background:** Cardiovascular disease (CVD) significantly impacts Alzheimer’s Disease and Related Dementia (AD/ADRD) mortality. South Carolina has a high incidence of CVD and dementia mortality. The aim of this study, therefore, was to examine the neurological causes of death and the leading causes of death in the South Carolina Alzheimer’s Disease Registry (SCADR). **Method:** Data from 2005–2018 were extracted from the SCADR using ICD-9 and ICD-10 codes. The top 10 leading causes of death (LCOD) were identified using death certificates. Some neurological causes of death were operationalized by combining related ICD codes, such as CVD_C (I219, I251, I500, I64) and chronic obstructive pulmonary disease (COP_C), (J449, C349), and $\chi^{2}$ was used to compare socio-demographic characteristics and mortality. Adjusted hazard ratios (aHR) and 95% confidence intervals (CI) were estimated using extended Cox Proportional Hazard modeling, adjusting for socio-demographic factors. **Results:** A total of 207,093 registry cases were included in the analysis. About 70% of cases had Alzheimer’s Disease (AD) diagnosis, and 40% of all cases were 85 years and older. The LCOD was CVD_C (13.4%). The risk of death among cases with vascular dementia (VaD) was 1.17 times the risk of death among those with AD (aHR: 1.172, 95% CI: 1.148–1.196). Among all deaths, cases with COP_C had a significantly higher likelihood of death compared to those with CVD_C (aHR: 1.06, 95% CI: 1.025–1.090). **Conclusions:** The study highlights CVD_C as the LCOD in frequency, with survival analysis indicating COP_C risk of death as significantly higher compared to CVD_C deaths. There is a need to prioritize CVD and lung-related comorbidity prevention, assessment, and management programs for individuals living with ADRD.

## 1. Introduction

All-cause mortality has been reported in the Global Burden of Disease (GBD) study [1]. In the US, dementia was the 6th leading cause of death in 2019 and 8th in 2021 (after the COVID-19 pandemic). Stroke and cerebrovascular diseases were reported as the second most prevalent cause of death (COD), while ischemic heart disease is the most prevalent COD [1].

The overall age-standardized death rate per 100,000 individuals is reported as 139 (121.3–173.3) globally, and 75.8 (56.7–114.7) in the US. Globally, the leading cause of death (LCOD) among individuals 60+ years of age attributable to disorders affecting the nervous system was stroke/cerebrovascular disease [2]. Alzheimer’s Disease (AD) was the second LCOD among all disorders affecting the nervous system. Although age-standardized deaths per 100,000 individuals decreased globally by 33.6% between 1990 and 2021 [2], this favorable trend was not found among individuals with AD, who showed an age-standardized death rate increase of 1.5%. The burden of disability measured by Disability Adjusted Life Years (DALYs) also increased slightly for AD (1.7%) while DALYs attributable to all disorders affecting the nervous system decreased by 27% [2]. These findings show that neurological conditions remain a major cause of global disease burden, and neurodegenerative diseases in particular have been refractory to scientific advances aimed at reducing both morbidity and mortality.

Although the age-standardized mortality associated with stroke/cerebrovascular disease has decreased by 34.9% over time, globally, dementia mortality has increased over this period [3]. Several authors have noted an association between a history of stroke/cerebrovascular disease and dementia morbidity and mortality [4,5,6]. It is, therefore, surprising that improvements in stroke/cerebrovascular disease mortality have not translated into a decreased mortality or disability due to AD. Further, many stroke/cerebrovascular disease risk factors, including hyperlipidemia, diabetes, hypertension, smoking, and sedentary lifestyle, are also positively correlated with dementia severity and mortality [7,8,9].

Conversely, among individuals diagnosed with AD/ADRD, there are positive correlations between stroke/cerebrovascular disease risk factors, imaging findings (large- and small-vessel strokes, and nonspecific microvascular disease), and mortality. For example, the apolipoprotein E4 allele is found in about 15% of individuals diagnosed with AD; it accelerates its course, while also predisposing to substantial intracranial hemorrhage risks associated with cerebral amyloid angiopathy [10,11]. Cerebrovascular diseases accelerate the progression of dementia severity, and clinically unrecognized sequelae such as cerebral white matter disease may produce cognitive impairments even among individuals not diagnosed with dementia syndrome [12,13]. The discrepancy between improving global stroke/cerebrovascular disease mortality and stagnant trends in AD/ADRD mortality/disability, thus, requires further research.

Identifying modifiable factors that contribute to AD/ADRD death is of major public health importance for many reasons. While disease-modifying therapies for AD/ADRD remain only modestly effective, optimal management of modifiable CODs such as stroke/cerebrovascular disease is a worthy focus; unfortunately, favorable public health interventions to control risk factors have improved age-standardized mortality without improving global AD/ADRD mortality. This suggests the need for an improved understanding of modifiable stroke/cerebrovascular disease risk factors that may contribute to AD/ADRD mortality. Recently, for example, exposure to environmental pollution from forest fires has been reported to increase AD/ADRD prevalence by 21% [14], adding environmental complexity to the many health factors that contribute to the intricate and bidirectional relationship between stroke/cerebrovascular disease and AD/ADRD. Although it makes sense that improving AD/ADRD mortality risks such as stroke/cerebrovascular disease risk factors should also improve morbidity and allowing affected individuals to enjoy better health and longevity, the stability of AD/ADRD prevalence in the setting of improved vascular mortality rates indicates that there is a gap in our understanding between mortality risks and AD/ADRD prevalence/morbidity.

Established in 1988, the South Carolina Alzheimer’s Disease Registry (Registry) is the oldest statewide dementia registry in the United States. Data collected is deidentified for research and public health purposes. The SC Registry is characterized as an “epidemiologic” Registry because it captures a reasonably representative sample of the SC population. For instance, approximately 23% of the 541,039 SC deaths over the reported study period (2005–2018) were captured by the Registry, according to a comparison of mortality statistics from the Centers for Disease Control WONDER database [15].

South Carolina (SC) is a state with one of the highest rates of stroke/cardiovascular disease and dementia mortality [16,17], and the Registry provides an ideal mechanism to examine the most common neurological causes of death (COD). The aim of this study was, therefore, to examine the LCOD, the relationships between stroke/cardiovascular disease, and AD/ADRD mortality, and to determine how LCOD may differ in the AD/ADRD categories.

## 2. Materials and Methods

### 2.1. Data Source

We collected data from the South Carolina Alzheimer’s Registry (Registry) from 2005 to 2018. Currently, the Registry includes data from over 377,000 individuals with AD/ADRD. The Registry captures International Classification of Disease codes (ICD-9 and ICD-10), medical claims data, and death certificate data to identify individuals diagnosed with AD/ADRD. Registrants are then classified into four dementia types, Alzheimer’s Disease (AD), Vascular Dementia (VaD), Mixed (AD plus VaD), and Other, based on the ICD-9 or ICD-10 codes assigned by their healthcare providers [18].

The present study reports leading causes of death based on death certificate data from 2005 through 2018. The year of death is automatically coded for all registrants, and the month of death was obtained from linking to death certificates. These death records also include up to 10 ICD-9/ICD-10 codes reflecting various diseases and disorders that contributed to each death. We excluded the most recent four years for which complete data are available to eliminate the confounding impact of COVID-19 on dementia mortality.

### 2.2. Exposure Assessment

The top 10 underlying causes of death in the Registry were extracted using the *International Classification of Diseases, 10th Revision (ICD-10)* [19]. Alzheimer’s disease is identified by code *G309*, dementia by code *F03*, atherosclerotic heart disease of native coronary artery without angina pectoris by code *I251*, chronic obstructive pulmonary disease (COPD), unspecified, by code *J449*, acute myocardial infarction, unspecified, by ICD code *I219*, Parkinson’s disease by ICD code *G20*, heart failure by ICD code *I500*, cerebrovascular disease/stroke (not specified as hemorrhage or infarction) by ICD code *I64*, malignant neoplasm of unspecified part of the bronchus or lung by ICD code *C349*, and pneumonia, unspecified organism, by ICD code *J189*. For this study, ICD codes *I219, I251*, *I500*, and *I64* were combined to include these cardiovascular and stroke/cerebrovascular disease categories (CVD_C); ICD codes *J449* and *C349* were combined into Chronic Obstructive Pulmonary Disease categories (COP_C) because of their shared anatomical focus, overlapping clinical features, and similar risk profiles in populations with ADRD [20,21,22]. The combination of ICD codes for research purposes has been previously reported [23]. For cases ascertained under ICD-9 codes, the Registry algorithm utilizes a crosswalk to match ICD-9 and ICD-10 diagnoses (see Appendix A).

### 2.3. Outcome Assessment

The total number of deaths in the Registry was calculated. Codes appearing on the death certificate were used to estimate the leading causes of death (LCOD) in the Registry for all registrants and for each of four diagnostic categories defined by the Registry ascertainment process; these diagnostic categories were Alzheimer’s disease (AD), Vascular Dementia (VaD), Mixed Dementia, and Other Dementia (Dementia in other medical conditions). The LCOD was stratified by diagnostic category, and overall LCOD statistics were also computed.

### 2.4. Time, Event, and Censoring

The follow-up period for the study was measured in months from the year of diagnosis or entry into the Registry (using 2005 as a baseline). Survival time was calculated as the difference between the year of death and the year of diagnosis or entry into the Registry. This was calculated in years and converted to months. An event was described as 1 if death occurred between 2005 and 2018 and set at 0 if the subject was alive or death occurred after 2018. Subjects with event 0, meaning subjects alive after 2018 or subjects alive at the last known follow-up, were censored.

### 2.5. Covariates

The covariates in the analysis included age, sex, and race. These have been reported to be associated with AD/ADRD [24,25].

### 2.6. Inclusion and Exclusion Criteria

The analysis was restricted to data from the South Carolina Alzheimer’s Registry. Subjects whose age of entry into the Registry was between 50 and 110 years from 2005 to 2018 were included. Participants with missing data on the year of entry, missing year of death, or whose year of death was earlier than their year of diagnosis or entry into the Registry were excluded.

### 2.7. Statistical Analysis

All statistical analyses were performed using SAS version 9.4 software (SAS Institute, Cary, NC, USA). A $\chi^{2}$ test was used to compare participants’ socio-demographic characteristics and mortality in the Registry, and these were presented as percentages. The hazard ratios (HR) and 95% confidence intervals for the association between the LOCD, diagnostic category, and the risk of death in the Registry while adjusting for age, sex, and race were estimated using a fully adjusted multivariate extended Cox regression model. Covariates were selected for the Cox model using a stepwise method selection with entry (SLE) and stay (SLS) criteria of 0.05 with *p*-values < 0.05. The proportional hazards assumptions were tested using the supremum test for all covariates. The supremum test is used to assess whether the hazard assumptions are violated or satisfied in a Cox proportional hazards modelling [26]. A *p*-value < 0.05 suggests that the assumptions have been violated for a specified covariate. The covariates violating the proportional hazard assumption were included in an extended Cox proportional hazards model.

## 3. Results

### 3.1. Demographic Characteristics

A total of 207,093 participants were included in the Registry, between 2005 and 2018, and a total of 122,252 deaths were recorded. During this same period, the Centers for Disease Control and Prevention’s CDC WONDER database reports a total of 541,039 SC deaths (including individuals with and without an ADRD diagnosis) within the same age range captured by the Registry. This indicates that the AD/ADRD deaths in SC captured within the Registry represent 22.6% of all SC deaths in the CDC WONDER database within the same period and same age range.

Most registrants (69.7%) had an Alzheimer’s disease (AD) diagnosis, and the category with the smallest percentage was Mixed Dementia (4.0%). About 40% of the registrants were 85 years and above. Most of the registrants with AD were 85 years and above, whereas most patients with VaD, Mixed, or Other Dementia classifications were between 75–84 years. Additionally, more than half of the registrants (60.3%) were females, and 69.9% were classified as White. All sociodemographic characteristics showed a significant association with mortality (*p*-values < 0.05). (Table 1).

### 3.2. Leading Causes of Death

Table 2 illustrates the top 10 LCODs, and Table 3 shows that the most common, operationalized underlying cause of death in the Registry was CVD_C (13.4%). Alzheimer’s disease accounted for 13.3% of all deaths among registrants, while unspecified dementia accounted for another 12.1% of deaths. Also, about 6.2% of all deaths were attributed to COP_C, a composite pulmonary category exclusive of pneumonia (1.7%) and Parkinson’s (2.5%). The other leading causes of death accounted for 50.9% of all deaths.

For the dementia-specific diagnosis, Alzheimer’s disease unspecified accounted for about 16.4% of all Alzheimer-specific deaths, 4% of VaD, 11.6% of Mixed, and 4.8% of Other deaths among registrants. CVD accounted for 12.6% of Alzheimer’s deaths, and 18.8%, 16.7%, and 12.9% of VaD, Mixed, and Other dementia-specific deaths, respectively. Table 3 also lists the causes of death for all deaths by each of the four dementia classifications among registrants. Table 2 and Table 3 summarize the death distributions by the top 10 LCODs and the most common LCODs (operationalized) in the Registry.

**Figure 1:**
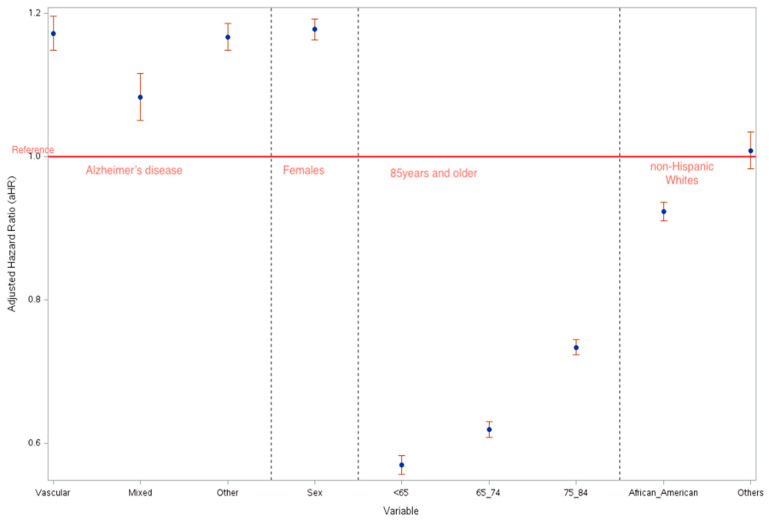
Adjusted-hazard ratio by the diagnostic type, sex, age group, and race.

Figure 1 shows the adjusted hazard ratios (adjusted for age, sex, and race) for each dementia classification and key demographics. The risk of death among registrants with vascular dementia was 1.17 times the risk of death among participants with Alzheimer’s diagnosis after adjusting for sex, age, and race (aHR: 1.172, 95% CI: 1.148–1.196). The aHR for death among individuals with Mixed Dementia was 1.08 times the risk of registrants classified as Alzheimer’s (aHR: 1.083, 95% CI: 1.051–1.116), and the aHR for death among individuals with other dementia was 1.17 times the risk of registrants with Alzheimer’s disease (aHR: 1.167, 95% CI: 1.148–1.186) (Figure 1).

### 3.3. Survival Analysis

Table 4 summarizes the association between the LCOD and the risk of death in the Registry according to dementia type, sex, age group, and race. Among all deaths, participants with chronic obstructive pulmonary disease categories (COP_C) had the highest risk of death, slightly higher than the risk of death from CVD_C, after adjusting for sex, age group, and race (aHR: 1.057, 95% CI: 1.025–1.090). For all deaths, the risk of dying from AD, Dementia, and Parkinson’s disease was significantly lower compared to the risk of dying from CVD_C. Among registrants with AD, the likelihood of a COP_C cause of death was significantly higher compared to CVD_C cause of death (aHR: 1.096, 95% CI: 1.056–1.138), while for other LCODs, including AD, Dementia, and Parkinson’s disease, the risk of death was significantly lower compared to CVD_C (see Table 4).

Similarly, for registrants classified as VaD, Mixed, and Other Dementia, the likelihood of AD, Dementia, or Parkinson’s disease cause of death was significantly lower than a CVD_C cause of death. Although the likelihood of Pneumonia (unspecified) as a cause of death was higher than CVD_C cause of death among VaD and Other deaths, it was not statistically significant (aHR: 1.089, 95% CI: 0.927–1.278, and 1.029, 95% CI: 0.917–1.155, respectively).

Registrants with Other dementia had a higher risk of CVD_C cause of death compared to AD, Dementia, and Pneumonia causes of death. The likelihood of Pneumonia (unspecified) and COP_C cause of death, among other dementia deaths, was higher than CVD_C; however, the hazard ratios were not statistically significant (see Table 4).

## 4. Discussion

CVD_C is the leading cause of death (by absolute frequency) in the Registry. However, in the survival analysis, the risk of COP_C cause of death was higher than CVD_C among all deaths. Additionally, males have a higher risk of death than females, and African American individuals have a lower risk of death compared to White individuals in the Registry.

Our findings on CVD_C LCOD are consistent with United States national mortality statistics, which consistently list cardiovascular diseases as the LCOD, followed by cancer, with AD/ADRD listed as the sixth LCOD [27]. In the Registry, AD and unspecified dementia (AD/ADRD) combined account for nearly twice as many deaths as CVD_C, although CVD_C is still the single LCOD. Registry case ascertainment methods rely on ICD-10 codes and systematically select individuals with an AD/ADRD diagnosis. Therefore, the fact that AD/ADRD is nearly twice as likely an LCOD than CVD_C may reflect a sampling/selection bias because the Registry specifically examines individuals with an AD/ADRD diagnosis.

Survival analysis accounts for survival time and adjusts for time-varying risks from entry into the Registry until death. There are shared risk factors between ADRD and COP_C, such as, chronic obstructive pulmonary disease (COPD) [28]. The interaction of COPD and ADRD has been suggested to be associated with poor quality of life, increased hospitalization, and overall, a decrease in survival [29,30]. Co-morbidity in ADRD patients, including those with COPD, may be treated with anticholinergics, a potentially inappropriate medication class. Potentially inappropriate medications increase the risk of hospitalization, infections, and death among people living with ADRD, may worsen respiratory function, and increase the risk of death [31,32]. COPD exacerbation and brain pathology studies showed that it can lead to physiological changes related to infection, respiratory failure, and even myocardial injury [33].

People living with ADRD with a comorbid COPD diagnosis are more likely to have other chronic conditions, including gastroesophageal reflux disease (GERD), also associated with acute COPD exacerbations [34]. This heightens their risk of complications, such as aspiration and pneumonia. The presence of aspiration, dysphagia, and aspiration pneumonia, which are linked to both COPD and ADRD, reduces survival rates and elevates mortality risk [35].

The relative importance of CVD_C was evaluated by examining the prevalence of CVD_C diagnoses among the four dementia types represented in the Registry. Not surprisingly, CVD_C causes of death are highest among individuals classified as vascular dementia and lowest among Registrants classified as AD. This may represent a type of “circular reasoning”. In other words, individuals who have many cardiovascular risks, including stroke and microvascular pathology on brain imaging, may be more likely to be classified as VaD than AD. Mixed Dementia (AD plus VaD) may be diagnosed when there are slightly fewer obvious CVD_C diagnoses present, and this view is supported by the intermediate prevalence of CVD_C in this subgroup.

The adjusted hazard ratios in this study demonstrate that men have a higher mortality risk than women. The various aspects of sex differences in ADRD have been previously reported, including physiological changes during development and changes associated with aging among men and women [36]. The lifetime risk of AD at age 45 years was higher in women than in men [17,37]. Men tend to have higher cardiovascular-related mortality risk than women and higher mortality risk following an ADRD diagnosis [38,39]. Our findings of increased mortality risk in men than women corroborated previous studies examining the predictors of survival among people living with Alzheimer’s disease [40]. In a systematic review to explore the predictors of survival among people living with ADRD, Zheng et al. reported that males had a higher risk of death compared to their female counterparts [41]. Although the Framingham study reported higher mortality risk in females than males, they suggested survival bias may have accounted for their observation [42]. Additionally, females tend to have longer survival times than males, thus resulting in lower mortality risk when compared to males [43]. A longer survival time may contribute to a lower hazard ratio/risk because the risk of death is distributed over an extended period, thereby reducing the likelihood of death occurring within this period. The differences in results presented above may be attributed to different study designs, variations in inclusion and exclusion criteria, and historical differences in case identification and diagnoses.

Our study found that mortality risk for African American individuals was lower (protective) compared to their White counterparts. Our findings are consistent with previous research, which found lower mean survival time in White individuals and an overall higher mortality rate among white individuals than black or African American individuals [44,45,46,47]. Many factors may account for this observation. First, education is associated with better cognitive reserve, which may delay cognitive impairment and, thus, delay an ADRD diagnosis. Older adults with at least 16 years of formal education have a higher mean cognitive reserve with less evidence of biomarkers corresponding to Alzheimer’s disease [48]. Secondly, longer years of formal education (at least 16 years) improve mental functioning, and higher education also influences a person’s lifestyle choices, such as, for example, diet, physical activity, and informed decisions through lifelong learning and the environment they live in. Older black adults in the Southern part of America experienced segregation, limited opportunities, and unfavorable educational policies, and attended underfunded schools [49,50]. Lower education affects cognitive reserve as well as performance on assessments commonly used to identify ADRD; hence, individuals with low educational attainment, such as the Black or African American participants in the present study, may be diagnosed earlier than White individuals. Also, diagnostic intensity may vary regionally and could alter the likelihood of receiving an AD/ADRD diagnosis for some demographic groups, depending on the diagnostic intensity within their community [51]. The lower mortality risk (hazard risk) among Black individuals may be due to differences in the likelihood of receiving an AD/ADRD diagnosis as well as support or care after diagnoses [52,53,54], particularly in populations with high prevalence of serious comorbidities at the time of diagnosis [50]. African Americans or Latinos are less likely to be put in nursing homes compared to their White counterparts and also have a higher risk of comorbidities such as hypertension, diabetes, and smoking, among others. Factors such as these may differentially impact overall survival post-diagnosis [53,54].

Conversely, a higher risk/rate of mortality for minority populations has been reported in previous research [55]. Several factors, including differences in the etiology of dementia, have been suggested, and African American and Asian individuals are more likely to die from vascular causes than Alzheimer’s disease [56]. Also, close contact among individuals in nursing homes increases the risk of pneumonia and upper respiratory infection, and population densities within long-term care environments may differ based on economic considerations. In our study, the mortality risk for Black or African Americans was lower than Whites. Disparities in care in medical settings may increase the risk of death among Blacks compared to their White counterparts with an ADRD diagnosis [57].

Our findings underscore the critical link between cardiovascular and pulmonary health outcomes among older adults living with AD/ADRD. In South Carolina, the ‘*Take Brain to Heart*’ campaign initiative [58], which emphasizes making healthy lifestyle choices, prioritizing mental health, investing in quality sleep, and improving nutrition, is an encouraging step in the right direction. This initiative highlights the importance of preserving brain function and aligns with the implications of our study, which may help address the cardiovascular and pulmonary disease mortality among older adults living with AD/ADRD.

### Strengths and Limitations

The relatively large sample size provided by the Registry, the consistent case ascertainment methodology employed since 1988, and the Registry’s representation of over 22% of all SC deaths are all important strengths of this study. Thus, the study has sufficient statistical power to identify many important and subtle effects. The combination of ICD codes also improves the comparability to existing literature. Sociodemographic factors were adequately adjusted to minimize unmeasured confounding. Additionally, we excluded the most recent years to reduce the confounding effect of COVID-19 deaths in this study.

The study, however, had some limitations. It may still be prone to residual confounding, as other sociodemographic factors, including educational status, were not able to be adjusted for or assessed. The reliability of dementia subtype diagnosis may be limited because patients in ‘Other dementia types’ may have vascular, mixed, or Alzheimer’s disease. Additionally, selection and observer bias may likely be present since different physicians may use varying criteria to designate dementia diagnoses, and to report the cause of death for patients with a comorbidity, thereby affecting generalizability. The differences in the timing and likelihood of diagnosis (diagnostic intensity) may vary among population subgroups, as may the risk for developing specific types of ADRD (e.g., stroke or myocardial infarction among men and minority populations). These differences influence their risks of dying after diagnosis. Additionally, the combination of ICD codes may limit the interpretation of cause-specific mortality risk.

## 5. Conclusions

To the best of our knowledge, this is the first study to utilize a population-based Alzheimer’s Registry to relate the most common neurological conditions, including CVD, COPD, Parkinson’s disease, and pneumonia, to AD/ADRD mortality risk and to determine how LCOD differs in the leading AD/ADRD diagnoses. This study showed that CVD_C is the LCOD in the Registry and, therefore, supports the need for physicians to assess and develop cardiovascular-specific prevention and management programs for people living with AD/ADRD, including resources in nursing homes and specialized facilities to support cardiovascular care. Although more people with AD/ADRD died from CVD_C, the COP_C mortality risk was higher among all deaths in the Registry compared to CVD_C-related deaths. Therefore, our findings suggest that it is also important to prioritize the prevention, assessment, and management of comorbidities such as lung cancer, pneumonia, and chronic obstructive pulmonary disease.

Prior work has shown that Black individuals in the Registry survive longer than their White counterparts in the Registry [59]. The crude mortality rate for Black individuals in the Registry is higher than White individuals. Understanding regional variations in LCOD and AD/ADRD comorbidity may reveal geographic targets for public health interventions that may reduce excess mortality among individuals with AD/ADRD, particularly Black individuals. Additionally, there is a need for public health interventions to address those LCOD that are amenable to intervention (e.g., smoking cessation for chronic obstructive pulmonary disease and hypertension/cholesterol control for cardiovascular diseases).

These findings have important implications for future research and offer testable hypotheses for future Registry studies. First, public health interventions that target education may delay the development of an AD/ADRD diagnosis [60]. If low educational attainment is a key factor in early-stage diagnosis of AD/ADRD, this may explain increased longevity in the Registry among those individuals who are potentially educationally disadvantaged. Second, improving both formal education and public health interventions aimed at cardiovascular and lung diseases will likely have downstream effects on mortality risk, particularly among Blacks in the Registry. Future work should examine these hypotheses.

## Figures and Tables

**Table 1:** Sociodemographic characteristics and death in the Registry.

Characteristic	Total	AD	VaD	Mixed	Other	*p*-Value *
All Deaths (%)	122,252 (100)	85,233 (69.72)	11,874 (9.71)	4935 (4.04)	20,210 (16.53)	<0.0001
Age Group						<0.0001
<65	9908 (8.66)	4644 (5.83)	1503 (13.50)	262 (5.74)	3499 (18.39)	
65–74	18,381 (16.07)	11,258 (14.14)	2382 (21.39)	802 (17.56)	3939 (20.70)	
75–84	40,368 (35.30)	28,628 (35.95)	3864 (34.70)	1783 (39.04)	6093 (32.02)	
>85	45,713 (39.97)	35,106 (44.08)	3387 (30.41)	1720 (37.66)	5500 (28.90)	
Sex						<0.0001
Male	47,490 (39.73)	30,725 (36.95)	5183 (44.68)	2009 (40.90)	9573 (48.19)	
Female	72,034 (60.27)	52,420 (63.05)	6418 (55.32)	2903 (59.10)	10,293 (51.81)	
Race						<0.0001
White	85,427 (69.88)	61,420 (72.06)	7104 (59.83)	3341 (67.70)	13,562 (67.11)	
African American/Black	27,247 (22.29)	17,108 (20.07)	3849 (32.42)	1287 (26.08)	5003 (24.76)	
Other	9578 (7.83)	6705 (7.87)	921 (7.76)	307 (6.22)	1645 (8.14)	

* *p*-values were derived from the *χ*^2^ test. Note: The sum of deaths by sex, race, or age group may not equal the overall total due to missing data on sex, race, and age group in some records.

**Table 2:** Top 10 leading causes of death in the Registry.

Cause of Death	All Deaths N (%)	Alzheimer’s N (%)	VaD N (%)	Mixed N (%)	Other N (%)
Alzheimer’s disease, unspecified	17,133 (13.25)	15,104 (16.36)	475 (4.01)	581 (11.57)	973 (4.84)
Unspecified dementia	15,686 (12.13)	12,382 (13.41)	920 (7.76)	655 (13.05)	1729 (8.59)
Atherosclerotic heart disease of native coronary artery	5545 (4.29)	3844 (4.16)	637 (5.38)	242 (4.82)	822 (4.09)
Cerebrovascular disease/stroke	5055 (3.91)	3067 (3.32)	902 (7.61)	378 (7.53)	708 (3.52)
Chronic obstructive pulmonary disease	4913 (3.80)	3513 (3.81)	443 (3.74)	140 (2.79)	817 (4.06)
Acute myocardial infarction	3637 (2.81)	2479 (2.69)	403 (3.40)	123 (2.45)	632 (3.14)
Parkinson’s disease	3252 (2.52)	1302 (1.41)	124 (1.05)	49 (0.98)	1777 (8.83)
Heart Failure/I500	3037 (2.35)	2219 (2.40)	285 (2.41)	96 (1.91)	437 (2.17)
Malignant neoplasm of part of bronchus or lung	3086 (2.39)	2012 (2.18)	296 (2.50)	80 (1.59)	698 (3.47)
Pneumonia, unspecified	2208 (1.71)	1558 (1.69)	185 (1.56)	91 (1.81)	374 (1.86)
Other	65,739 (50.85)	44,820 (48.56)	7179 (60.59)	2585 (51.49)	11,155 (55.44)

**Table 3:** Operationalized leading causes of death in the Registry.

Cause of Death	All Deaths N (%)	Alzheimer’s N (%)	VaD N (%)	Mixed N (%)	Other N (%)
CVD_C	17,274 (13.36)	11,609 (12.58)	2227 (18.79)	839 (16.71)	2599 (12.92)
Alzheimer’s disease, unspecified	17,133 (13.25)	15,104 (16.36)	475 (4.01)	581 (11.57)	973 (4.84)
Unspecified dementia	15,686 (12.13)	12,382 (13.41)	920 (7.76)	655 (13.05)	1729 (8.59)
COP_C	7999 (6.19)	5525 (5.99)	739 (6.24)	220 (4.38)	1515 (7.53)
Parkinson’s disease	3252 (2.52)	1302 (1.41)	124 (1.05)	49 (0.98)	1777 (8.83)
Pneumonia, unspecified	2208 (1.71)	1558 (1.69)	185 (1.56)	91 (1.81)	374 (1.86)
Other	65,739 (50.85)	44,820 (48.56)	7179 (60.59)	2585 (51.49)	11,155 (55.44)

**Table 4:** Association between operationalized LCOD and the risk of death in the Registry in relation to CVD_C deaths.

LCOD	Risk of Death				
	All Deaths aHR * (95% CI)	AD aHR (95% CI)	VaD aHR (95% CI)	Mixed aHR (95% CI)	Other aHR (95% CI)
Alzheimer’s Disease	**0.790 (0.771–0.810)**	**0.812 (0.790–0.836)**	**0.777 (0.693–0.871)**	**0.768 (0.680–0.868)**	**0.830 (0.764–0.901)**
Dementia	**0.905 (0.883–0.928)**	**0.918 (0.891–0.945)**	**0.911 (0.834–0.995)**	**0.838 (0.747–0.940)**	0.968 (0.905–1.036)
COP_C	**1.057 (1.025–1.090)**	**1.096 (1.056–1.138)**	0.920 (0.838–1.011)	0.953 (0.808–1.124)	1.041 (0.969–1.117)
Parkinson’s Disease	**0.856 (0.818–0.895)**	**0.805 (0.751–0.863)**	**0.797 (0.644–0.986)**	**0.703 (0.499–0.989)**	**0.894 (0.833–0.960)**
Pneumonia, unspecified	0.989 (0.942–1.040)	0.977 (0.920–1.037)	1.089 (0.927–1.278)	0.975 (0.768–1.239)	1.029 (0.917–1.155)
Sex					
Males vs. Females	**1.219 (1.196–1.243)**	**1.235 (1.208–1.263)**	**1.145 (1.071–1.225)**	**1.190 (1.082–1.308)**	**1.159 (1.104–1.218)**
Age Group					
<65 vs. 85 and above	**0.495 (0.474–0.516)**	**0.474 (0.448–0.501)**	**0.554 (0.491–0.625)**	**0.446 (0.354–0.562)**	**0.503 (0.461–0.549)**
65–74 vs. 85 and above	**0.582 (0.565–0.599)**	**0.565 (0.546–0.585)**	**0.652 (0.593–0.717)**	**0.620 (0.540–0.711)**	**0.587 (0.548–0.629)**
75–84 vs. 85 and above	**0.707 (0.692–0.721)**	**0.693 (0.677–0.710)**	**0.760 (0.702–0.821)**	**0.755 (0.682–0.836)**	**0.735 (0.695–0.778)**
Race					
African American vs. White	**0.881 (0.861–0.902)**	**0.855 (0.831–0.880)**	**0.909 (0.844–0.978)**	**0.775 (0.694–0.866)**	0.994 (0.936–1.055)
Others vs. White	**1.054 (1.011–1.098)**	1.045 (0.995–1.098)	1.062 (0.921–1.223)	0.816 (0.658–1.013)	**1.123 (1.021–1.236)**

* aHRs and 95% CIs were derived from an extended Cox proportional hazards modelling. Bolded aHRs and 95% CIs are statistically significant.

**Figure A1:**
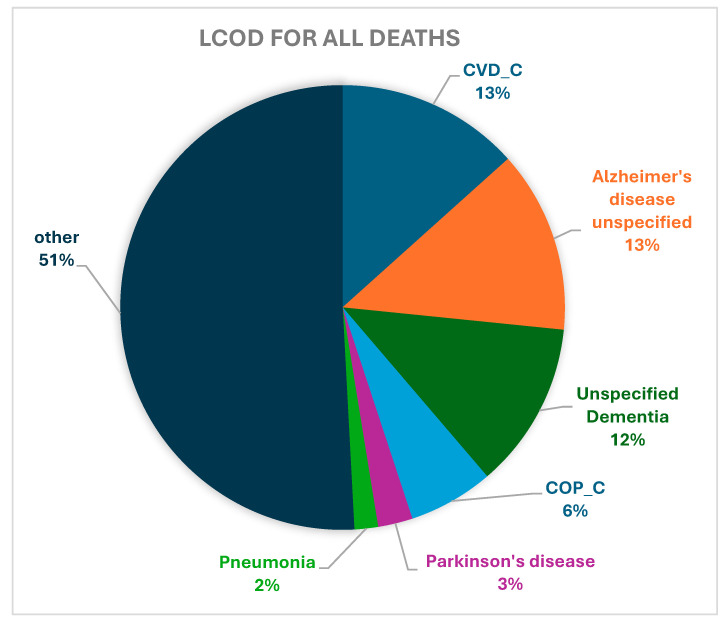
LCOD for all deaths in the Registry.

**Figure A2:**
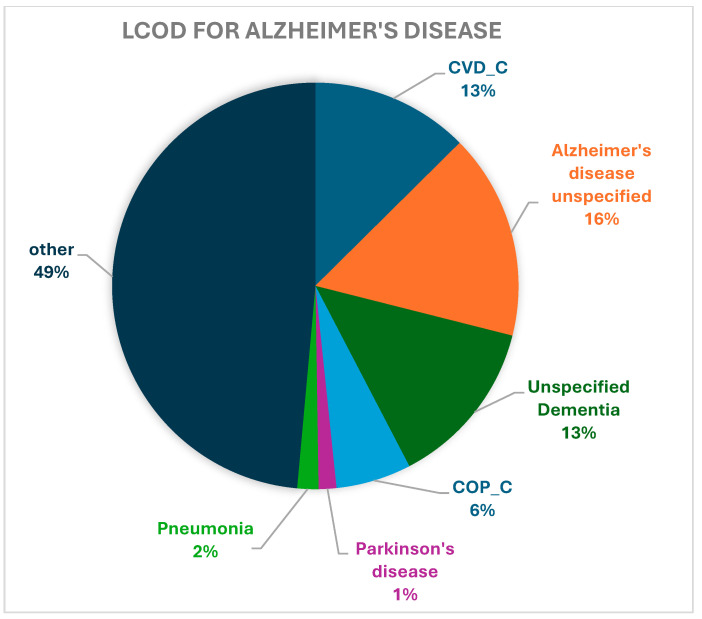
LCOD for participants with Alzheimer’s diagnosis.

**Figure A3:**
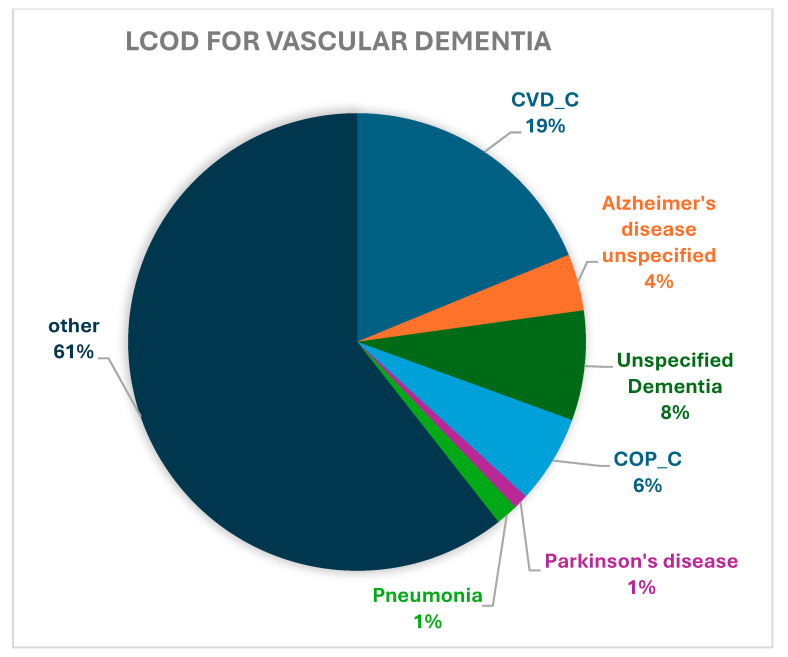
LCOD for participants with vascular dementia.

**Figure A4:**
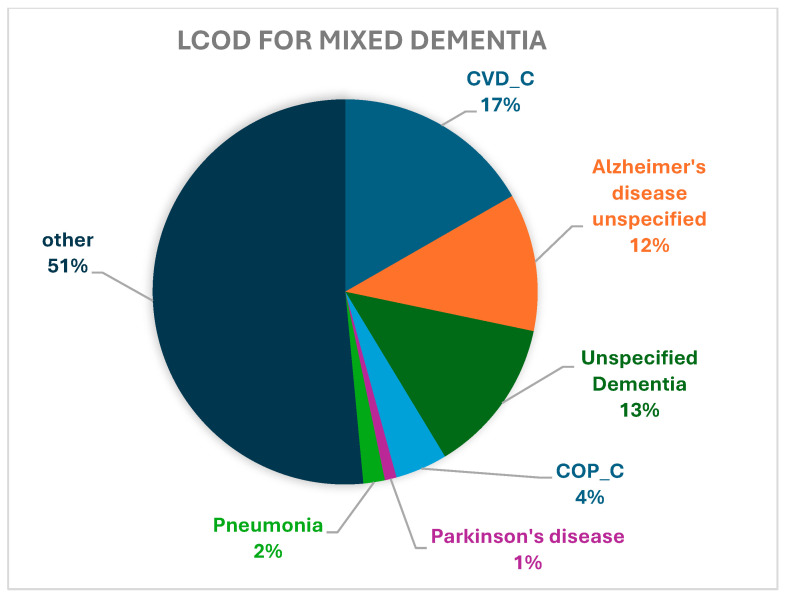
LCOD for participants with mixed dementia diagnosis.

**Figure A5:**
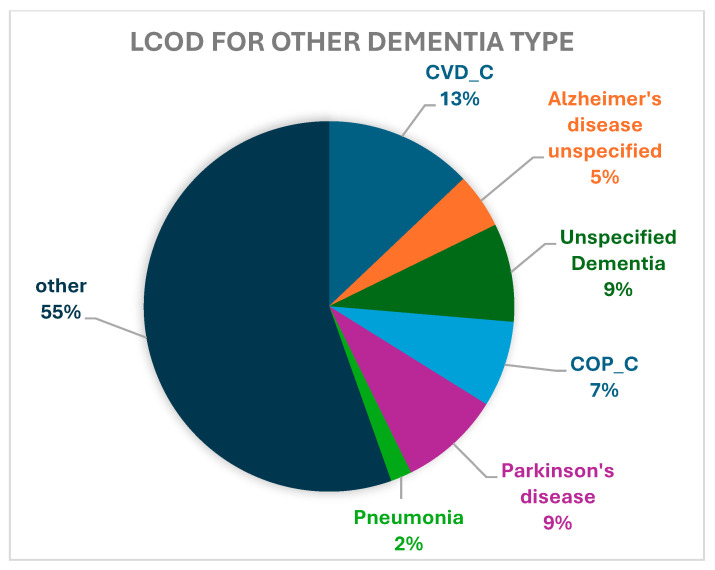
LCOD for participants with other dementia diagnosis.

## Data Availability

The data used for this study are subject to licenses and restrictions. Data request and access can be obtained at https://osa-sc.org/programs/alzheimers-disease-registry (last accessed on 25 May 2025).

## Abbreviations

COD	Cause of Death
LCOD	Leading Cause of Death
AD/ADRD	Alzheimer’s Disease/Alzheimer’s Disease or Related Dementias
CVD	Cardiovascular Disease
CVD_C	Cardiovascular Disease (operationalized)
COPD	Chronic Obstructive Pulmonary Disease
COP_C	Chronic Obstructive Pulmonary Disease (operationalized)
VaD	Vascular Dementia
SC	South Carolina
GERD	Gastroesophageal Reflux Disease
